# An International Year to Celebrate Nursing

**DOI:** 10.17533/udea.iee.v38n1e01

**Published:** 2020-02-26

**Authors:** María de los Ángeles Rodríguez Gázquez

**Affiliations:** 1 Editor of Investigación y Educación en Enfermería. Universidad de Antioquia, Colombia. Email: maria.rodriguezg@udea.edu.co Universidad de Antioquia Universidad de Antioquia Colombia maria.rodriguezg@udea.edu.co

On 16 May 2019, the 72^nd^ World Health Assembly designated 2020 as the *International Year of Nursing*. The decision was made by bearing in mind the substantial contribution made by this profession to the population’s health, and that this year marks the bicentennial of the birth of Florence Nightingale, one of the founders of modern nursing.(1)

The World Health Organization (WHO) states that, although nurses are half the health workers globally, it is necessary to add nine million of these professionals before 2030. The aforementioned seeks to reach the universal coverage in health, which is an indispensable goal to achieve the planet’s sustainable development objectives.(2)

The 2020 initiative: International Year of Nursing is supported by the WHO, the International Confederation of Midwives, the International Nurses Council, the campaign Nursing Now, and the United Nations Population Fund. Throughout the year and globally activities will be undertaken to celebrate the work of nursing and which show to public opinion the difficult conditions these professionals encounter, situations that must be improved by promoting investment to increase training and employment of this personnel as part of each country’s commitment with health for all.(2)

For 38 years, our journal, *Investigación y Educación en Enfermería*, has exalted the essential role of nurses in caring for individuals and communities, and will continue supporting efforts on the dissemination of knowledge to collaborate in the growth of this discipline, which, although young, presents a wealth of research as never seen before.(3) By this means our readers, authors, reviewers and members of the Editorial Committee are invited to, and not only for this year, write and publish about what they think, feel, and do for Nursing to help to make this world become a bit better each day.

Happy International Year of Nursing
